# Lipomatous Dural Metaplasia Associated With Chronic Ventricular Shunting: A Report of Two Cases

**DOI:** 10.7759/cureus.83902

**Published:** 2025-05-11

**Authors:** Pokhraj P Suthar, Shehbaz M Ansari, Golnaz Lotfian, Sarah Abdel-Hadi, Sumeet G Dua

**Affiliations:** 1 Department of Diagnostic Radiology and Nuclear Medicine, Rush University Medical Center, Chicago, USA

**Keywords:** chronic ventriculoperitoneal shunting, ct, dura, metaplasia, mri

## Abstract

Ventriculoperitoneal (VP) shunting is a critical intervention for managing hydrocephalus through cerebrospinal fluid (CSF) diversion. This report presents two rare and previously undocumented cases in which patients with long-standing VP shunts developed extensive dural fat accumulation, as evidenced by computed tomography (CT) and magnetic resonance imaging (MRI). These findings are highly suggestive of lipomatous dural metaplasia - a phenomenon that remains largely unaddressed in current medical literature. These cases are notable for their distinctive imaging features and the exclusion of alternative diagnoses, offering a compelling basis for further inquiry. By documenting these unique presentations, this manuscript aims to raise awareness and stimulate research into the pathophysiology, clinical implications, and diagnostic criteria of dural fat accumulation associated with chronic CSF shunting.

## Introduction

Ventriculoperitoneal (VP) shunting is crucial for draining cerebrospinal fluid (CSF) in various types of hydrocephalus [1]. However, long-term shunting entails complications such as over- or under-drainage, obstruction, disconnection/fracture, infections, and ventricular adhesions [1]. Several study groups have reported that the complication rate following VP shunt surgery falls within the range of 17% to 33% [2]. Post-shunting, diffuse dural abnormalities known as post-shunt meningeal fibrosis have been observed, resulting from an inflammatory response triggered by chronic subdural hemorrhage, leading to capillary ingrowth and fibroblast migration from the dura [3,4]. These changes are linked to recurrent hemorrhage and subdural fibrosis. Common imaging features include diffuse pachymeningeal enhancement, slit ventricles, subdural fluid collections, pituitary enlargement, and skull hyperosteosis [1]. In this report, we present two cases with diffuse accumulation of fat along the dural reflections on computed tomography (CT) and magnetic resonance imaging (MRI) - a finding never before reported. We believe that this rare imaging finding is a result of either fatty metaplasia of fibroblasts or meningeal stem cell adipocytic differentiation in response to chronic irritation. Comprehensive research is needed to fully comprehend this phenomenon’s complex pathophysiology and its potential impact on neurological health.

## Case presentation

Case 1

A 66-year-old male with a history of severe mental retardation, cerebral palsy, seizure disorder, VP shunt, Addison's disease, and hypothyroidism presented with recent-onset respiratory symptoms at a long-term care facility. Initial workup revealed a diagnosis of left lower lobe pneumonia. During the course of the patient’s hospital stay, he developed altered mental status with decreased responsiveness to commands or sternal rub, along with grimacing and localization of noxious stimuli in his head and neck. Notably, the eye examination showed downward eye deviation with intermittent rapid jerks to the right. Despite these neurological findings, his pupils were equal and reactive to light, and the oculocephalic and corneal reflexes were intact, with no facial asymmetry. Interestingly, he did not exhibit withdrawal or localization of noxious stimuli in his extremities but showed a brisk reflex response and triple flexion in both lower extremities. The patient was promptly treated for Addison's disease with hydrocortisone. Laboratory workup showed mild leukocytosis (11.3 K/uL; normal range 4.0-10.0 K/uL) and anemia (hemoglobin 9.1 g/dL; normal range 13.5-17.5 g/dL). Other routine laboratory results were within normal limits (Table 1).

**Table 1:** Laboratory results of Case 1. The patient exhibited mild leukocytosis and anemia. Other routine laboratory values were within normal limits. Reference ranges are included for context.

Parameter	Result	Reference Range	Interpretation
White Blood Cell Count	11.3 K/uL	4.0-10.0 K/uL	Mild leukocytosis
Hemoglobin	9.1 g/dL	13.5-17.5 g/dL	Anemia

**Figure 1:**
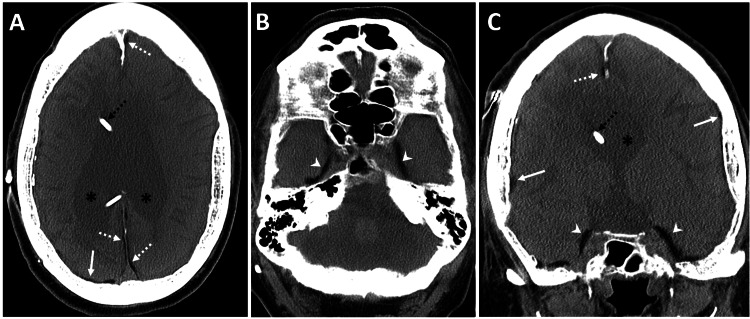
Diffuse dural fat deposition and cerebral atrophy in a patient with chronic VP shunting. A 66-year-old male with a medical history of altered mental status, in the context of known severe mental retardation, cerebral palsy, and a VP shunt. Unenhanced CT images of the brain, obtained with soft tissue settings - (A and B) axial and (C) reformatted coronal views - show diffuse atrophy of the brain parenchyma related to known cerebral palsy, with prominent bilateral lateral ventricles (black asterisks in A and C) and a right frontal approach VP shunt catheter (dashed black arrow in A and C). Incidentally, a diffuse dural fat collection (Hounsfield Unit -70) is seen along the falx cerebri (dashed white arrows in A and C), cerebral convexities (solid white arrows in A and C), and middle cranial fossa (white arrowheads in B and C), without sulcal or basal cistern fat densities. There is associated calvarial hyperostosis from chronic venous shunting. VP, ventriculoperitoneal; CT, computed tomography

**Figure 2:**
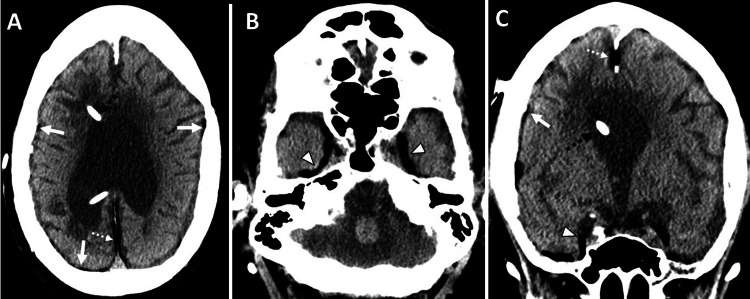
Brain soft tissue window images corresponding to Figure 1. (A and B) Axial and (C) reformatted coronal images show diffuse atrophy of the brain parenchyma, related to known cerebral palsy, with prominent bilateral lateral ventricles and a right frontal approach VP shunt catheter (dashed black arrow in A and C). Incidentally, a diffuse dural fat collection (Hounsfield Unit -70) is seen along the falx cerebri (dashed white arrows in A and C), cerebral convexities (solid white arrows in A and C), and middle cranial fossa (white arrowheads in B and C), without sulcal or basal cistern fat densities. There is associated calvarial hyperostosis from chronic venous shunting. VP, ventriculoperitoneal

A brain CT scan without contrast was performed to rule out acute intracranial abnormalities. The CT scan revealed moderate dilatation of the lateral ventricles and severe thinning of the corpus callosum, with a right frontal approach VP shunt in situ. Additionally, there was associated atrophy of the cerebral peduncles, brainstem, and cerebellar hemispheres. Incidentally, diffuse fat accumulation (Hounsfield Unit -70) was observed along the falx cerebri, cerebral convexities, and middle cranial fossa. A possible differential of ruptured dermoid was briefly considered, but closer inspection revealed that the distribution was entirely pachymeningeal, and the subarachnoid spaces - including the sulci and cisterns - were clear (Figures 1-2).

**Figure 3:**
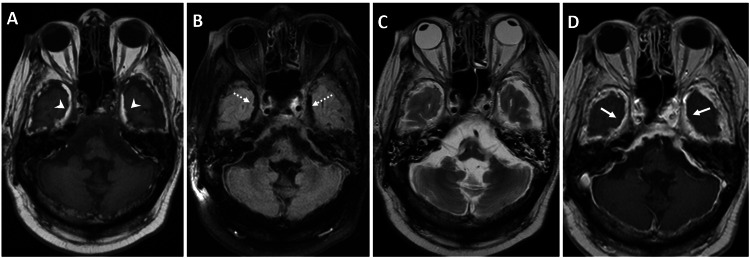
MRI characterization of dural fat deposition with corresponding signal changes and diffuse pachymeningeal enhancement. An MRI of the brain was performed in a 66-year-old male with a medical history of altered mental status, in the context of known severe mental retardation, cerebral palsy, and a VP shunt. (A) Axial T1-weighted unenhanced MRI brain images show dural T1 hyperintensity along the middle cranial fossa (white arrowheads), correlating with the fat density seen on the CT scan. (B) Axial FLAIR image shows a drop in signal corresponding to the dural T1 hyperintensity (dashed white arrows). (C) Axial T2-weighted MRI images show barely appreciable dural T2 hyperintensity along the middle cranial fossa, separable from the T2 hyperintense CSF signal. (D) Axial T1-weighted contrast-enhanced MRI brain image, after intravenous injection of 20 mL of gadoteridol (ProHance; Bracco, Milan, Italy), shows diffuse pachymeningeal dural enhancement (solid white arrows). MRI, magnetic resonance imaging; CT, computed tomography; FLAIR, fluid-attenuated inversion recovery; CSF, cerebrospinal fluid; VP, ventriculoperitoneal

**Figure 4:**
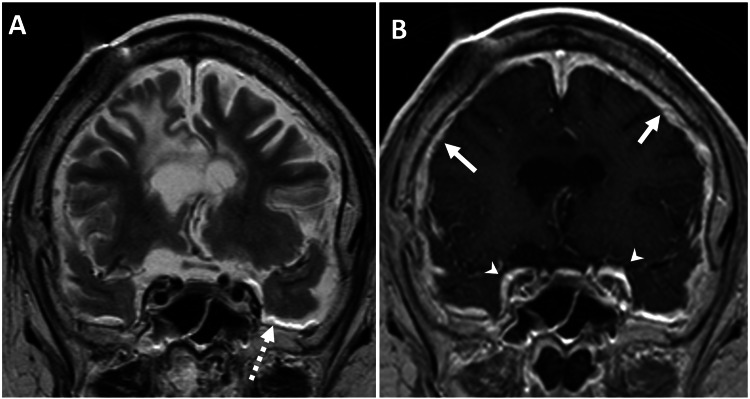
Coronal MRI demonstrating dural fat-fluid interface artifact and diffuse pachymeningeal enhancement in a patient with chronic VP shunting. An MRI of the brain was performed in a 66-year-old male with a medical history of altered mental status, in the context of known severe mental retardation, cerebral palsy, and a VP shunt. (A) Coronal T2-weighted MRI brain images show barely appreciable dural T2 hyperintensity along the falx, which is separable from the T2 hyperintense CSF signal. However, there is a chemical shift artifact related to the fat-CSF fluid interface along the bilateral middle cranial fossa (dashed white arrow). (B) Coronal T1-weighted contrast-enhanced MRI brain image, after intravenous injection of 20 mL of gadoteridol (ProHance; Bracco, Milan, Italy), shows diffuse pachymeningeal dural enhancement along the middle cranial fossa (white arrowheads) and cerebral convexities (solid white arrows). Additionally, there is diffuse atrophy of the brain parenchyma, with prominent bilateral lateral ventricles related to known cerebral palsy. A susceptibility artifact in the right frontal scalp is related to the VP shunt catheter reservoir. MRI, magnetic resonance imaging; VP, ventriculoperitoneal; CSF, cerebrospinal fluid

Diffuse thickening of the calvarium was also seen, consistent with long-term CSF shunting. A brain MRI was performed to further elucidate these findings. The MRI showed T1 hyperintensity along the dural reflections in the falx cerebri, over the cerebral convexities, and more prominently in the middle cranial fossa, corresponding to the fat density observed on the CT scan. Notably, the subarachnoid spaces were clear. The findings were confirmed on fluid-attenuated inversion recovery (FLAIR) and T2-weighted images, which showed signal dropout at the corresponding sites and a chemical shift artifact, respectively. Moreover, there was diffuse pachymeningeal enhancement along the falx cerebri, tentorium cerebri, cerebral convexities, and middle cranial fossa (Figures 3-4).

Case 2

A 46-year-old female presented to the Emergency Department (ED) with altered mental status. She had a long-standing history of VP shunting for obstructive hydrocephalus. The patient had a medical history of epilepsy, hydrocephalus, neurofibromatosis type 1, intellectual disability, and stroke. During the examination, the patient was obtunded and unresponsive to commands, and both eyes displayed a downward gaze deviation (setting-sun sign). Vital signs indicated tachypnea and mild hypertension. The neurological examination revealed sluggish bilateral pupillary responses and bilateral forced downward gaze deviation. Laboratory workup showed elevated arterial bicarbonate (25.4 mmol/L; normal reference range: 18-23 mmol/L), arterial CO₂ (26.2 mmol/L; normal reference range: 19-24 mmol/L), and venous lactate (3.6 mmol/L; normal reference range: 0.5-1.7 mmol/L). Additionally, mild leukocytosis (11.2 K/uL; normal range: 4.0-10.0 K/uL) was observed. However, the rest of the routine laboratory work was within normal limits, including a negative COVID-19 polymerase chain reaction (PCR) (Table 2).

**Table 2:** Laboratory results of Case 2. Laboratory analysis revealed elevated white blood cell count, arterial bicarbonate, arterial CO₂, and venous lactate. Remaining routine lab results, including COVID-19 PCR, were normal. PCR, polymerase chain reaction

Parameter	Result	Reference Range	Interpretation
White Blood Cell Count	11.2 K/uL	4.0-10.0 K/uL	Mild leukocytosis
Arterial Bicarbonate	25.4 mmol/L	18-23 mmol/L	Elevated
Arterial CO₂	26.2 mmol/L	19-24 mmol/L	Elevated
Venous Lactate	3.6 mmol/L	0.5-1.7 mmol/L	Elevated

**Figure 5:**
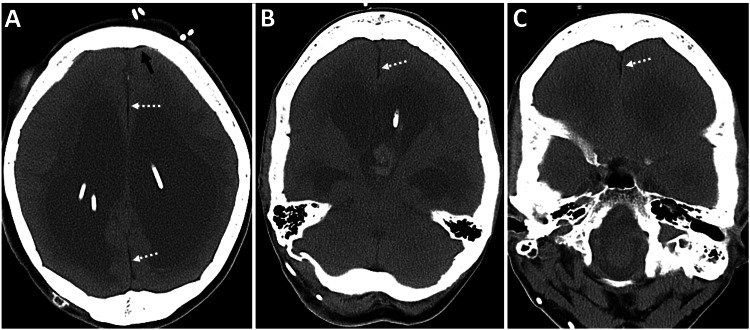
CT findings of obstructive hydrocephalus with intraventricular hemorrhage and incidental dural fat deposition in a patient with chronic shunting. A 46-year-old female presented with altered mental status, in the setting of multiple ventriculoperitoneal shunt placements for obstructive hydrocephalus. (A-C) Unenhanced axial CT images of the brain show marked supratentorial hydrocephalus with ventricular shunts and external ventricular drain catheters, along with intraventricular hemorrhage in the third ventricle and occipital horn of the left lateral ventricle. There is periventricular hypodensity, related to the transependymal flow of CSF. Incidentally, there is mild diffuse fat density along the dura in the interhemispheric falx cerebri (dashed white arrows in A-C) and along the cerebral convexity (solid black arrow in A), resulting from fatty metaplasia of the dura in the setting of chronic shunting. There is associated calvarial hyperostosis from chronic venous shunting. CT, computed tomography; CSF, cerebrospinal fluid

**Figure 6:**
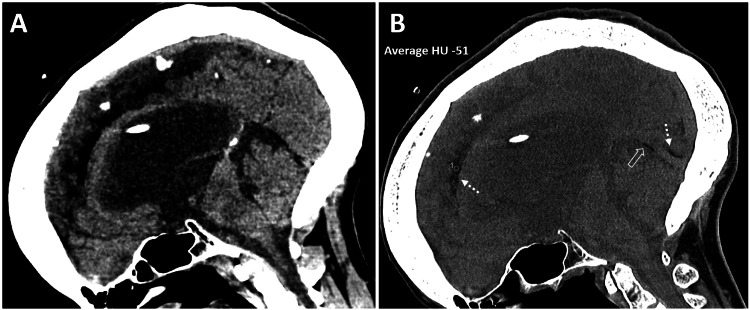
Sagittal CT depiction of supratentorial hydrocephalus, intraventricular hemorrhage, and dural fat deposition in chronic VP shunting. A 46-year-old female presented with altered mental status, in the setting of multiple VP shunt placements for obstructive hydrocephalus. An unenhanced sagittal reformatted CT image of the brain in (A) soft tissue algorithm and (B) bone algorithm with a soft tissue window setting shows marked supratentorial hydrocephalus with ventricular shunts and external ventricular drain catheters, along with intraventricular hemorrhage in the third ventricle. Incidentally, there is mild diffuse fat density (Hounsfield Unit -51) along the dura in the interhemispheric falx cerebri (dashed white arrows in B) and along the inferior sagittal sinus (open white arrow in B), best depicted in the bone algorithm with a soft tissue window setting. There is associated calvarial hyperostosis from chronic venous shunting. CT, computed tomography; VP, ventriculoperitoneal

CT of the brain without contrast was performed to rule out intracranial bleeding or other pathology. The CT scan revealed marked obstructive supratentorial hydrocephalus with ventricular shunts and external ventricular drain catheters, along with intraventricular hemorrhage in the third ventricle and occipital horn of the left lateral ventricle. There was periventricular hypodensity, likely related to the transependymal flow of CSF. Incidentally, a mild diffuse fat density was noted along the dura in the interhemispheric falx cerebri and inferior sagittal sinus (Figures 5-6). There was associated calvarial thickening from chronic venous shunting. In this case, an MRI was not performed. CSF examination yielded unremarkable results.

## Discussion

VP shunting aids in CSF drainage in various types of hydrocephalus [1]. The shunt can be placed temporarily before brain tumor removal or permanently in congenital malformations, such as aqueductal stenosis. Long-term shunting is associated with numerous complications, such as over- or under-drainage, obstruction, disconnection/fracture, infections, and ventricular adhesions [1]. Diffuse dural abnormalities post-shunting have been reported in the form of post-shunt meningeal fibrosis [3]. It is proposed that chronic subdural hemorrhage incites an inflammatory reaction with capillary ingrowth and fibroblast migration from the dura [3,4]. The former contributes to recurrent hemorrhage commonly observed with these chronic subdural collections and is the rationale behind middle meningeal artery embolization in such cases [4]. Fibroblast migration is responsible for eventual subdural fibrosis [3].

The known imaging features associated with VP shunting and intracranial hypotension typically include diffuse pachymeningeal enhancement, slit ventricles, subdural fluid collections, brain sagging, and pituitary enlargement. Chronic ventricular shunting is also associated with diffuse skull thickening. Herein, we present two cases in which we observed a diffuse dural fat collection on CT and MR imaging. The findings from CT scans and MRI support a plausible hypothesis of lipomatous dural metaplasia or stem cell differentiation. The diagnosis of this unusual entity can be challenging, given that fatty proliferation within the dura has never been reported before. Furthermore, both fat and fluid show low attenuation on CT, and small areas of fat often appear inseparable from CSF. In our case, we used attenuation measurements to confirm our diagnosis on CT. The detection of fat is relatively simpler on MRI, as seen in our case, with T1-weighted images being pivotal and FLAIR/T2 helping with confirmation, as in our case.

In the domain of medical literature, we put forth a conceivable supposition relating to lipomatous dural metaplasia, an issue yet to be investigated. The differential diagnosis of such diffuse lipomatous dural metaplasia could include age-related dural fat deposition or neoplastic lipomatous metaplasia, as seen in lipomatous meningioma and neurofibroma. Metaplastic differentiation of meningioma is rare and includes osseous, cartilaginous, myxoid, and lipomatous subtypes [5]. Intradural lipoma, though commonly associated with lumbar spinal dysraphism, has also been reported at the craniocervical junction [6]. These aforementioned differential diagnoses can easily be ruled out in our case, due to their focal nature.

Intracranial fat can also be seen with intracranial dermoid, which, when ruptured, can show multifocal intracranial subarachnoid fat droplets [7]. Intracranial pneumocephalus can be confused with fat, but a quick change to a negative window level and high window width (commonly known as the lung window) can help in differentiation. Air in pneumocephalus has a high negative Hounsfield value (-1000 HU), compared to adipose tissue (-100 HU) [8].

Focal fat deposition in the dura mater is not uncommon and was first reported in 1950 by Balo [9]. Subsequently, various authors have studied focal fat deposition in different regions of the dura mater and dural venous sinuses [10]. The most common regions include the cavernous sinus, torcula herophili, straight sinus, inferior sagittal sinus, and transverse sinus, in decreasing order of incidence [10]. These focal fatty infiltrations are considered part of the aging process, but the underlying mechanisms of these changes remain not fully understood. Additionally, diffuse fatty differentiation of the dura mater has not been previously reported, to the best of our knowledge. Based on the molecular biology discussed above, we propose that this could be related to the metaplastic potential of fibroblasts or adipocytic differentiation of meningeal stem cells as a response to chronic irritation. Similar metaplastic potential of dural multipotent cells has been proposed to be responsible for falx cerebri ossification [11]. These areas of ossification can display fatty marrow development, like bone marrow elsewhere, mimicking focal dural lipomatous metaplasia [11].

The adult meninges consist of a variety of cell types, including fibroblasts and neural stem cells [12]. Under certain stimuli, these cells can undergo transdifferentiation, transforming into different types of tissues like bone, fat, or cartilage [12]. Such transformations may occur in response to various types of central nervous system (CNS) injuries, tumors, or age-related processes [5,10]. Typically, the metaplastic response in these cases is focal. In this report, we present two cases of diffuse lipomatous metaplasia of the dura mater in patients with long-term VP catheters. The meninges comprise fibroblasts, mast cells, pericytes, smooth muscle cells, immune cells, telocytes, and endothelial cells. Studies have suggested the presence of subsets of undifferentiated stem cells in adult meninges [12]. Meningeal mast cells contribute to inflammatory responses with cell factors IL-6 and CCL7, while fibroblasts produce cytokines in response to trauma and injury and can differentiate into cartilage, fat, and other cell types [12]. Recent literature also suggests that adult meninges may contain neural stem/progenitor cells, usually dormant but activated following CNS injury, though the stem cell potential of the meninges is still under study [13].

**Figure 7:**
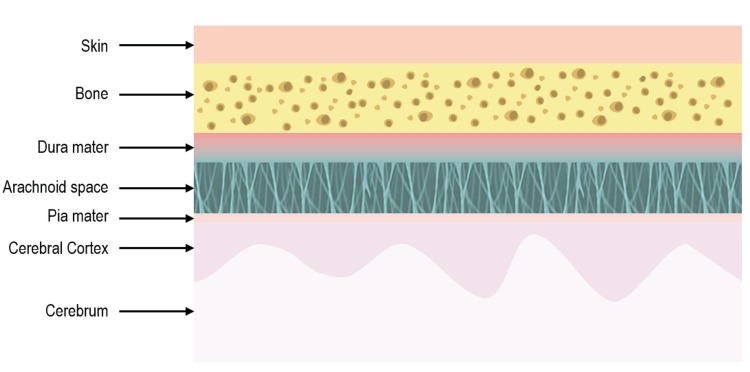
Illustration demonstrates layers of meninges. Image credit: Sarah Abdel-Hadi

**Figure 8:**
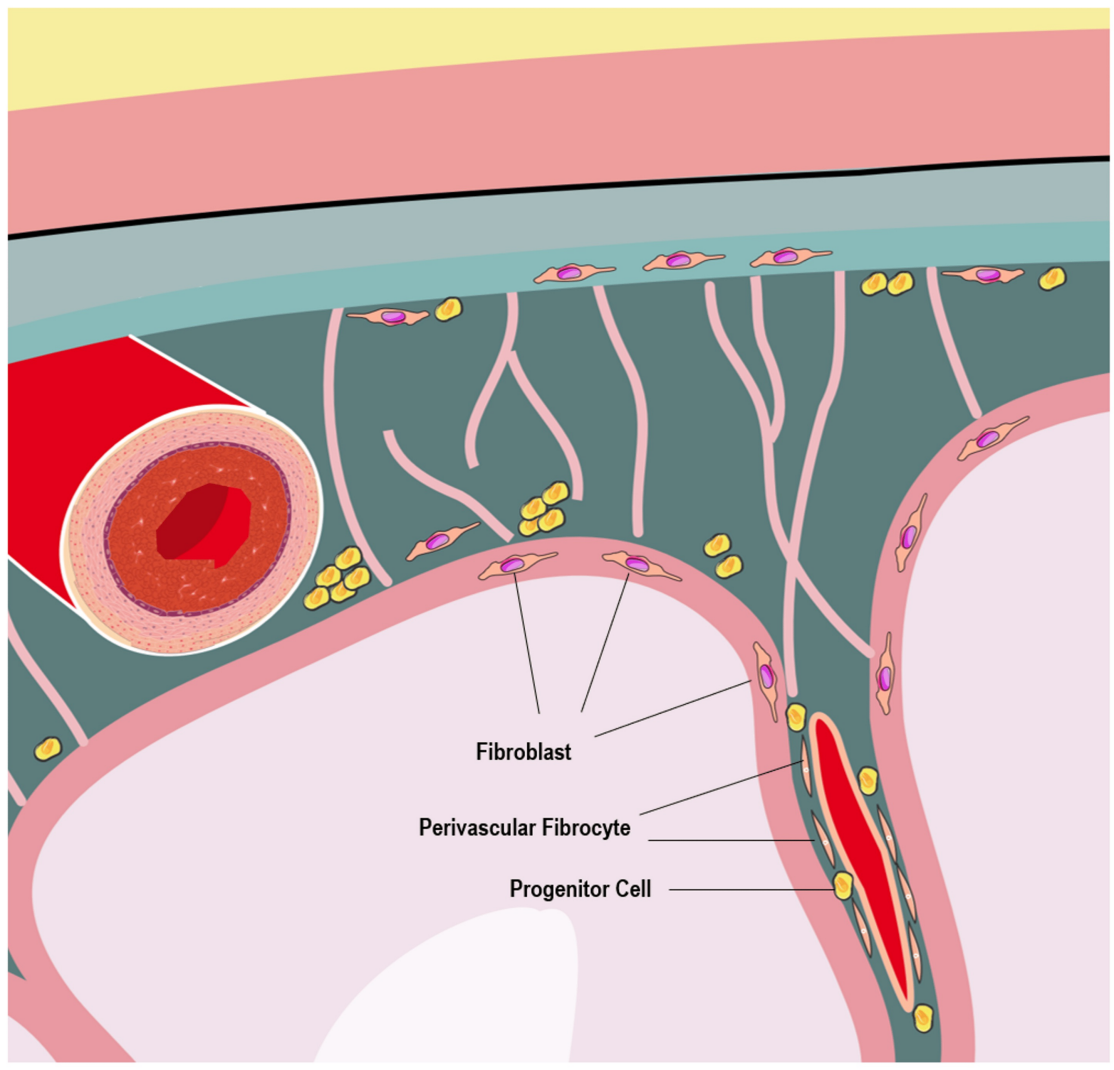
Illustration demonstrates distribution of neural progenitor cells, fibroblasts, and fibrocytes across meninges layers. Image credit: Sarah Abdel-Hadi

Fibroblasts produce various cytokines and growth factors and can differentiate into different cell types. They can be found in perivascular spaces, meninges, and the choroid plexus. Throughout the three layers of the meninges (pia mater, arachnoid mater, and dura mater), fibroblasts are present. CNS fibroblast lineage tracing studies reveal that meningeal fibroblasts in the midbrain, hindbrain, and spinal cord originate from the somatic and cephalic mesoderm, while those in the forebrain derive from the neural crest [13]. The inflammatory responses of meningeal mast cells and fibroblasts, along with fibroblasts' differentiation potentials, suggest the underlying mechanism of lipomatous metaplasia: CNS injury can trigger a proinflammatory response, leading to fibroblast differentiation and possible neural stem/progenitor cell differentiation, resulting in lipomatous metaplasia (Figures 7-8). Lipomatous metaplasia has been documented in multiple neoplasms, including melanocytic nevi, neurofibromas, and some appendage tumors [14]. Severe chronic inflammation, noted in the superficial dermis, is believed to contribute to this phenomenon [14].

Further research is needed to comprehensively understand the pathophysiology behind diffuse fatty differentiation of the dura mater, and related conditions like lipomatous metaplasia.

## Conclusions

VP shunting remains a cornerstone in the management of hydrocephalus, but long-term shunt placement can give rise to rare and underrecognized complications. This report presents two previously undocumented cases of extensive dural fat accumulation in patients with longstanding VP shunts, with imaging findings highly suggestive of lipomatous dural metaplasia - a phenomenon not addressed in current medical literature. The distinctive radiologic appearance and exclusion of alternative diagnoses support the notion of a novel pathological process, potentially driven by chronic CSF pressure alterations, dural irritation, or a metaplastic response to inflammation. Recognizing this entity is essential to avoid misinterpretation as more serious conditions, such as lipomatous tumors or infiltrative disease, and to guide appropriate clinical management. These cases underscore the need for heightened clinical awareness and further investigation into the prevalence, pathophysiology, and clinical impact of dural adipose transformation associated with chronic shunting. By documenting these unique presentations, this report aims to stimulate research that will expand our understanding of dural biology, refine diagnostic criteria, and ultimately improve the care of patients with complex CSF diversion histories.
